# Supplementary material for: Continuous flash suppression responses in mouse visual cortex: Stimulus laterality and anesthesia effects
Source: iScience. 2026 Jul 7;29(7):116628. doi: 10.1016/j.isci.2026.116628 (PMC13356775; doi:10.1016/j.isci.2026.116628)
Supplement: Document S1. Figures S1–S9 [file mmc1.pdf]

**iScience, Volume *29***

# Supplemental information

# Continuous flash suppression responses in mouse visual cortex: Stimulus laterality and anesthesia effects

**Mathis Bassler, Lilian Emming, Christopher J. Whyte, Gerjan Huis In 't Veld, Mototaka Suzuki, and Cyriel M.A. Pennartz**

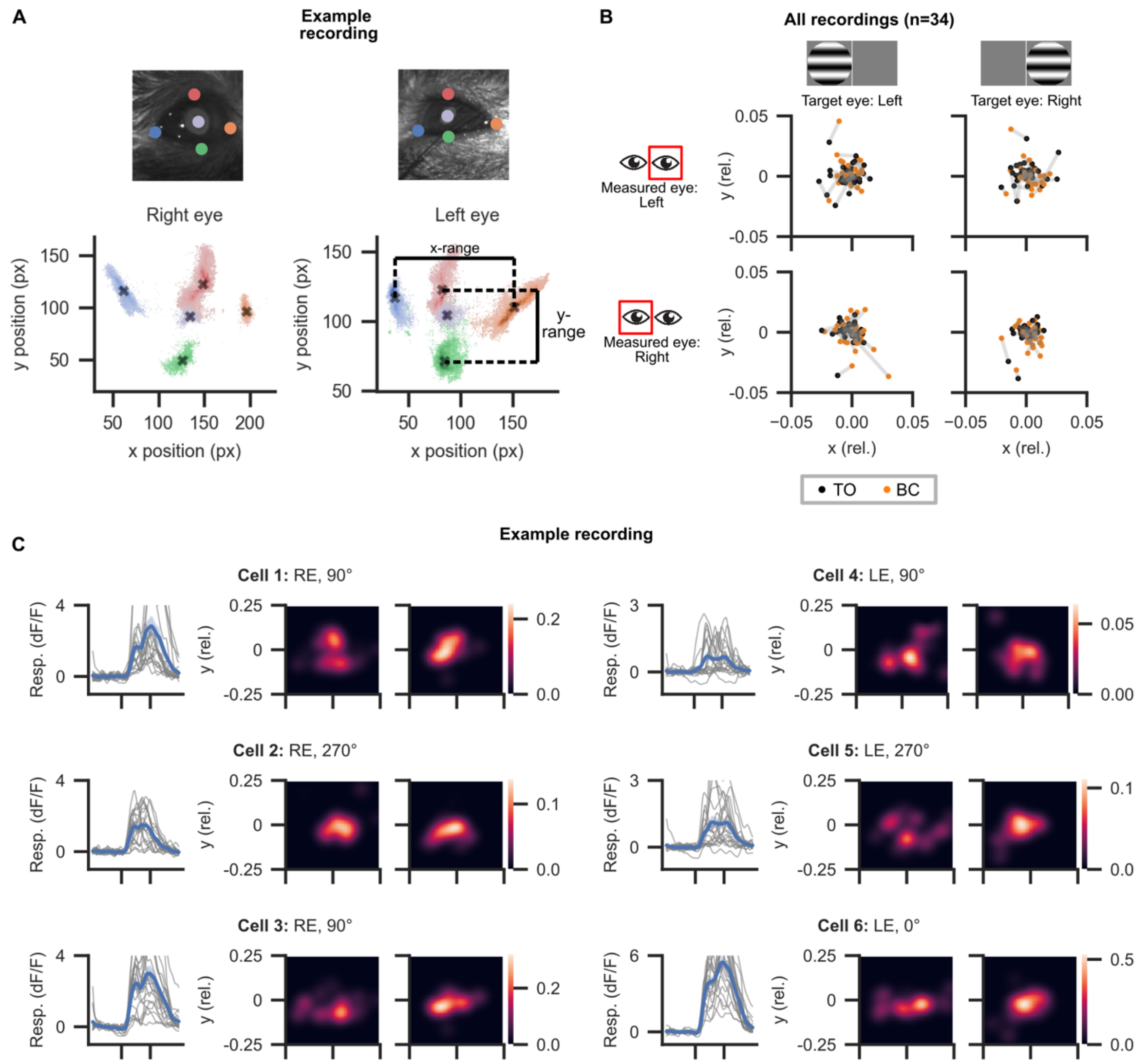


**Supplementary figure 1. Mouse pupil positions vary little during binocular stimulation.**
(A) Determination of pupil center position relative to eye corners. Pupil center and eye corner position values in pixels for the example recording session shown in Figure 1. Top: images of the two eyes annotated with keypoints. Bottom: 2D histograms of keypoint positions over the whole recording. Histogram colors match those of the keypoints. Crosses indicate the median keypoint positions within this session. Median pupil center position as well as eye width (x-range) and eye height (y-range) were used to scale pupil center coordinates relative to the eye (see Methods).
(B) Mouse pupil positions are similar across target-only (TO) and binocular conflict (BC) trials and cluster narrowly around the default pupil position. Median pupil positions during target-only (TO) and binocular conflict (BC) trials of 34 recording sessions (seven out of 8 mice) of the simultaneous-onset awake experiment. Each dot represents the median pupil position within one recording over 80 trials of a condition. Grey lines connect BC and TO medians of the same recording session. In this scaling system, an x-coordinate of 1 means the pupil center shifted away from its median position by half of the eye width (see Methods). rel.: relative to eye size (as developed in A). RE: right eye, LE: left eye. Overall, pupil positions within a recording during TO and BC conditions closely center around the median position.
(C) Neuronal responses are dependent on pupil positions. Responses of the six example neurons from Figure 1 to their preferred grating stimulus. The preferred grating stimulus is indicated on top of each plot. There are three panels per cell. (Left panel) Average response (blue) with SEM

(shaded blue) as well as individual responses for every stimulus repeat (n = 20, grey). (Middle panel) 2D heatmap of the neuronal response in relation to the eye position. Per trial (n = 20 trials), the average response during 1s stimulus presentation was logged in a 2D grid of the pupil position from -0.25 to 0.25. Afterwards, the grid was smoothed with a Gaussian kernel (sigma = 0.034 relative units). Note that dark regions may either indicate absence of neuronal firing at this pupil position or absence of sampling by the pupil.

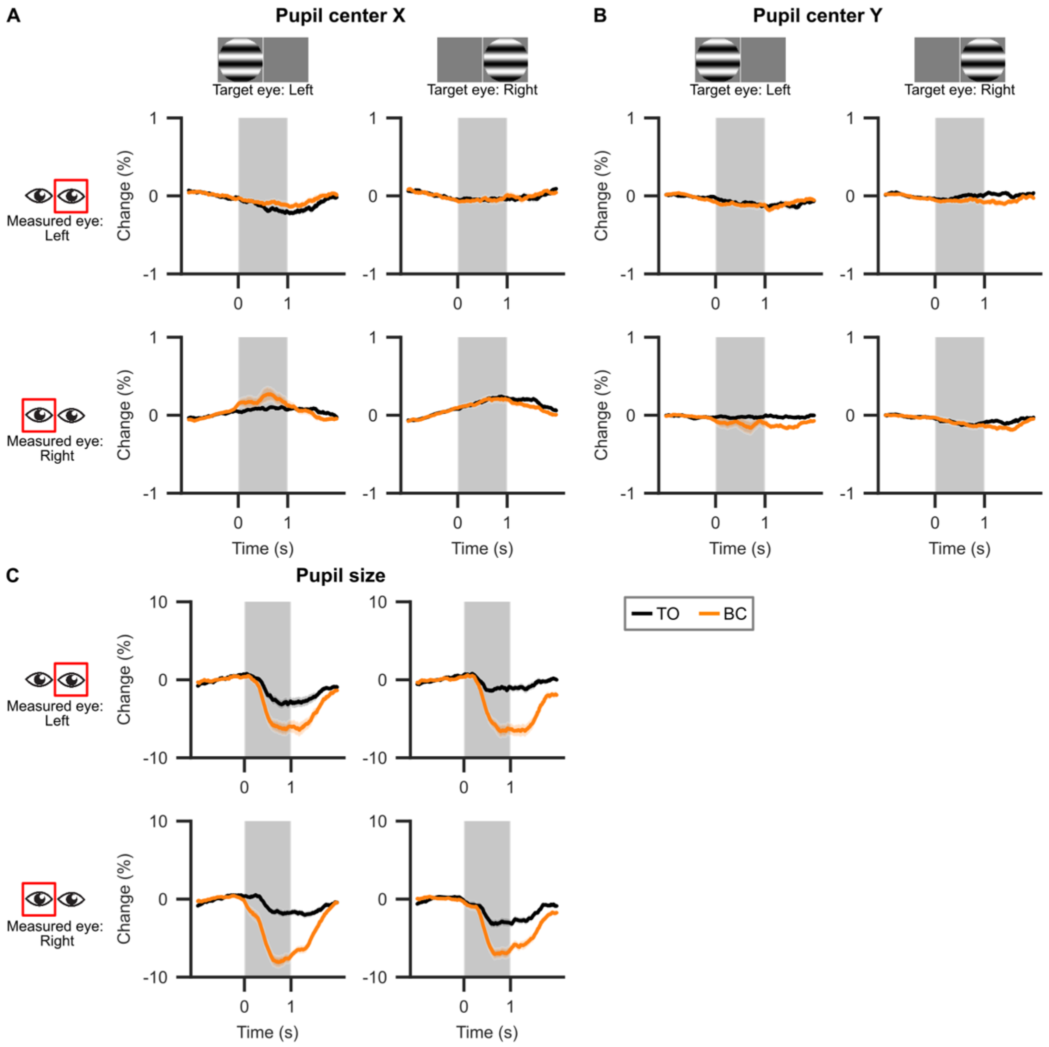


**Supplementary figure 2. Stimulus presentation elicits only minor and symmetric pupil position changes during the awake simultaneous-onset experiment.**

(A) Target-only (TO) and binocular conflict (BC) trials evoked only minor (and similar pupil position) changes. Change of x-position of pupil center during stimulus presentation compared to x-position during baseline (1s before stimulus onset). Changes are plotted separately for target displayed on the left or right eye (columns) as well for left and right pupil (rows). Averaged over n = 34 recordings (from seven out of eight mice). Shaded region around lines indicates standard error of the mean. Shaded grey region indicates stimulus presentation period. TO = target-only, BC = binocular conflict.

(B) Like (A) but now for Y-position of pupil center.

(C) Like (A) but now for pupil size. Pupil constriction upon stimulus onset was higher for BC than TO trials but similar for both eyes.

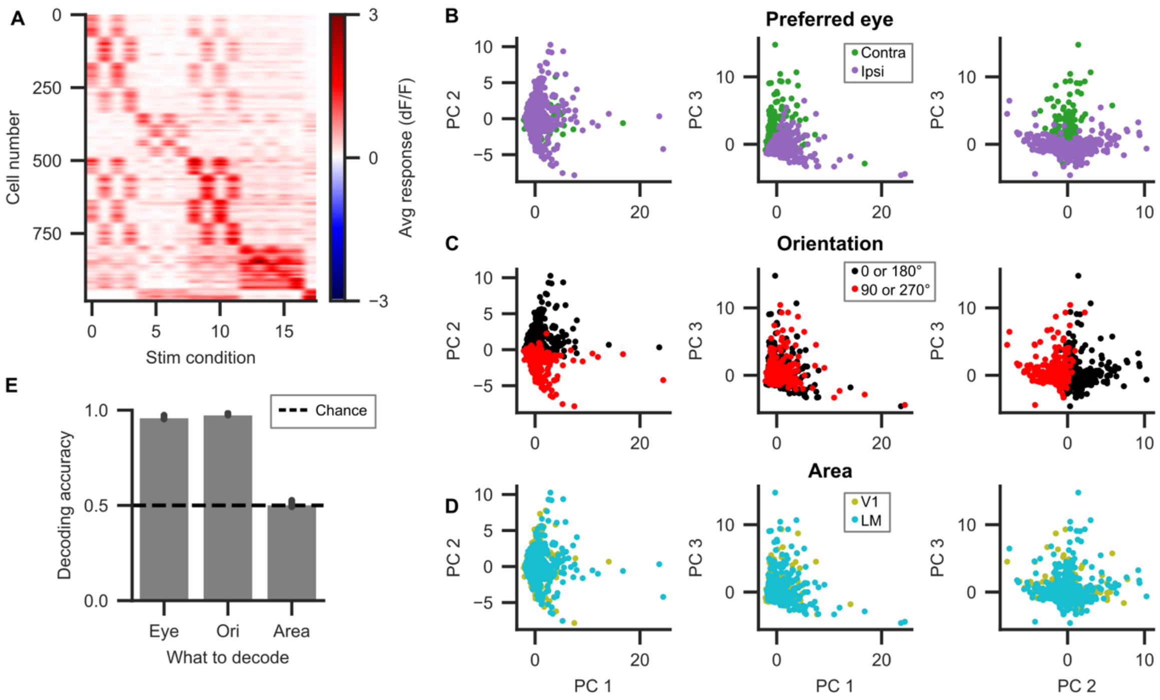


**Supplementary figure 3. V1 and LM cells are indistinguishable by linear classification.**

(A) Average responses of all cells in the awake simultaneous-onset experiment to all 18 stimulus conditions (eight monocular targets, eight binocular conflict displays, two monocular masks). Cells were sorted by condition with highest response for visualization purposes.

(B) Scatterplots of neuronal response patterns (i.e. the average responses during stimulus presentation for all 18 stimulus conditions) after transformation by PCA. Each dot represents a cell. Dots are colored based on the eye of origin of the preferred target. PC = Principal component. Contra = preferred target on contralateral eye, Ipsi = preferred target on ipsilateral eye.

(C) Same as (B) but now colored by preferred orientation.

(D) Same as (B) but now colored by brain area.

(E) Linear support vector machine decoding cannot distinguish V1 and LM cells above chance from their response patterns. Accuracy of decoding cell characteristics from their average responses to all stimulus conditions (depicted in A) using a linear support vector machine classifier. Eye = Eye of origin of preferred target (depicted in B). Ori = Orientation of preferred target (depicted in C). Area = Brain area (depicted in D). Dotted line indicates chance level decoding (50%).

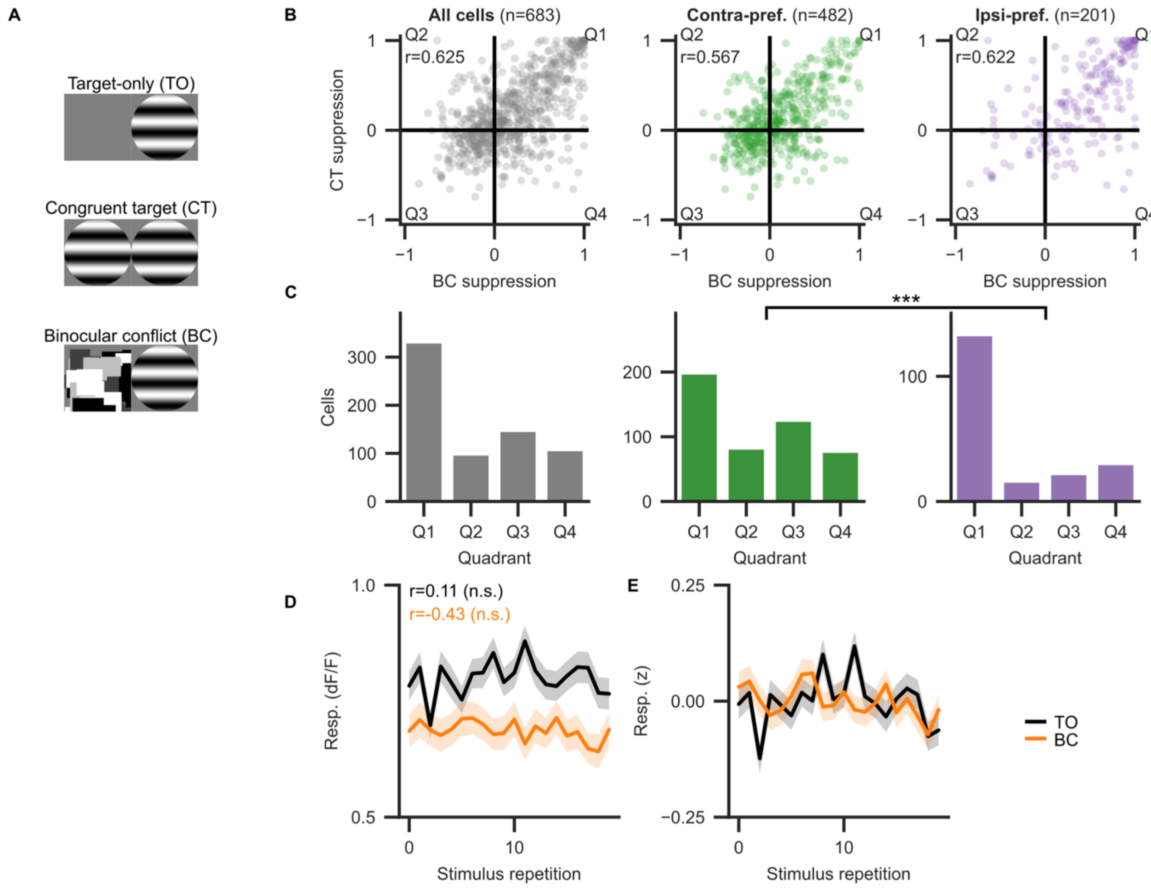


**Supplementary figure 4. Congruent stimulation evokes suppression in ipsi-preferring cells similar to binocular conflict; response amplitudes do not vary with stimulus repetitions.**
(A) In a subset of recordings (n = 26), mice (five out of eight) were presented with target-only (TO) and binocular conflict (BC) conditions as well as a control condition consisting of two congruent targets (CT).
(B) For a subset of cells from Figure 2 (n = 683/1004), we calculated suppression scores for BC and CT conditions. Suppression scores were generally well correlated. r = Pearson correlation coefficient. All correlations were significant ($p < 0.001$). Q1/2/3/4 = quadrant 1/2/3/4.
(C) Cells preferring ipsilateral targets generally exhibited lower responses to both BC and CT trials as compared to TO trials which suggests that the mechanism underlying BC response suppression is similar to that behind CT response suppression. We divided cells into four groups that are represented by the four quadrants in B. These groups indicated whether cell responses tended to be suppressed or enhanced (as compared to monocular (TO) responses) in BC and CT conditions (Q1: suppressed in both, Q3: enhanced in both, Q2: enhanced in BC, suppressed in CT, Q4: suppressed in BC, enhanced in CT). *** : $p < 0.001$, Chi-Square test between quadrant counts of contra- and ipsi-preferring cells.
(D) Average response (dF/F) of all 1004 cells to TO and BC trials per stimulus repetition. *r*: Pearson correlation coefficient between average population response and stimulus repetition count. n.s.: not significant. TO responses did not correlate with stimulus repetition (CI95% for r = [-0.35, 0.53], p = 0.643). BC responses tended to decrease with stimulus repetition although this effect was not significant (CI95% for r = [-0.73, 0.01], p = 0.058).
(E) Same as D but now z-scored by the average response of each cell in each condition.

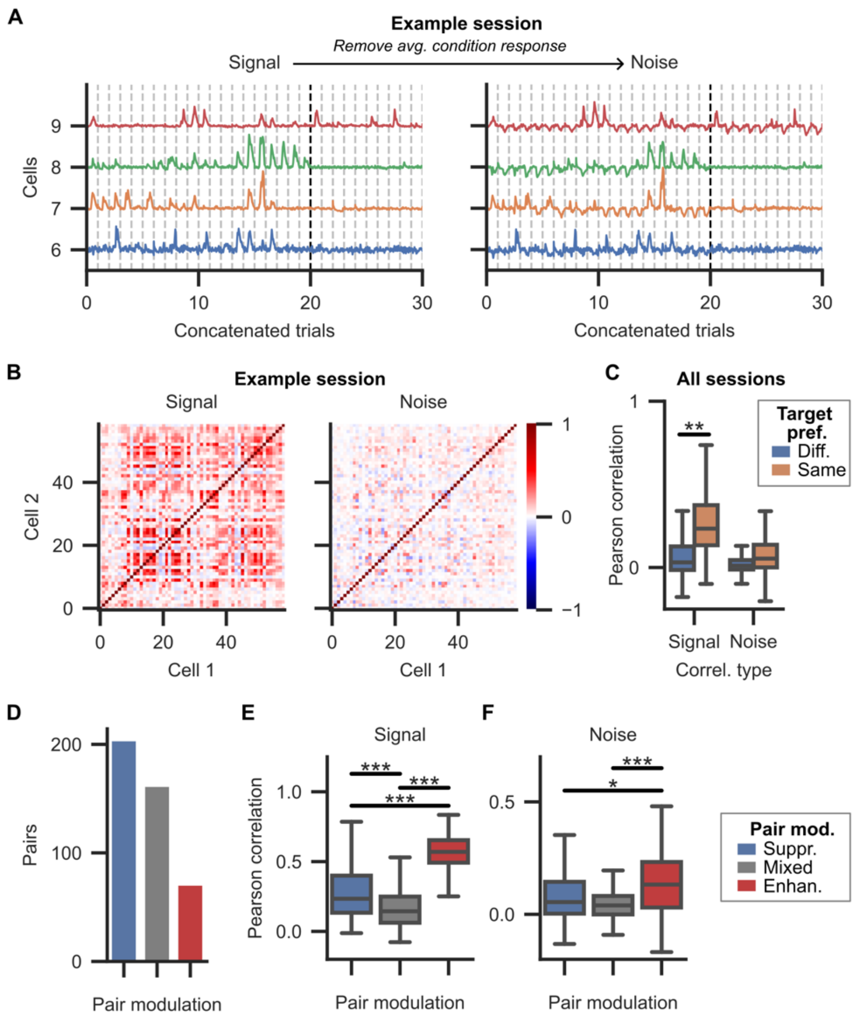


**Supplementary Figure 5: Neurons with enhanced responses to CFS stimuli also exhibit high noise correlations amongst each other.**
(A) Fluorescence traces of an example session of the simultaneous-onset experiment before and after subtracting condition response averages. As is custom in noise correlation analysis, the traces before average subtraction are referred to as “signal” while those after average subtraction are referred to as “noise”. For visualization purposes, only data from the first 30 trials and four neurons are shown here. Grey dashed lines indicate trial starts, black dashed lines indicate the transition to a new condition (every 20 trials).
(B) Signal and noise correlation matrices of the example session shown in B. Here and in the following, correlations are measured with the Pearson coefficient.
(C) Signal and noise correlations of cell pairs of all recording sessions of the simultaneous-onset experiment. Overall, we extracted 14,284 cell pairs (see Methods). Of those, 2,445 pairs had the same preferred target (17%). Signal correlations exceeded noise correlations only when cell pairs with the same target preference were considered. ** : $p < 0.01$, post-hoc Tukey HSD after two-factor ANOVA.
(D) Counts of cell pairs with the same preferred target that where either both suppressed (blue), both enhanced (red), or mixed suppressed/enhanced (grey; cf. Fig 2F for statistical assessment of CFS modulation). Note that for this panel and panels E and F, unaffected cells were excluded from the analysis. We find comparatively few pairs of enhanced cells (n = 71 pairs from 17 sessions in 6 mice), which is not surprising given that less than 15% of cells were enhanced (cf. Fig 2F).
(E, F) For cell pairs with the same target preference, neuron pairs in which both cells exhibit enhanced responses to CFS stimuli have not only higher signal but also higher noise correlations than other neuron pairs. This suggests a common source of variability in these cells. *** : $p < 0.001$,

** $p < 0.01$, post-hoc Tukey HSD after two-factor ANOVA.

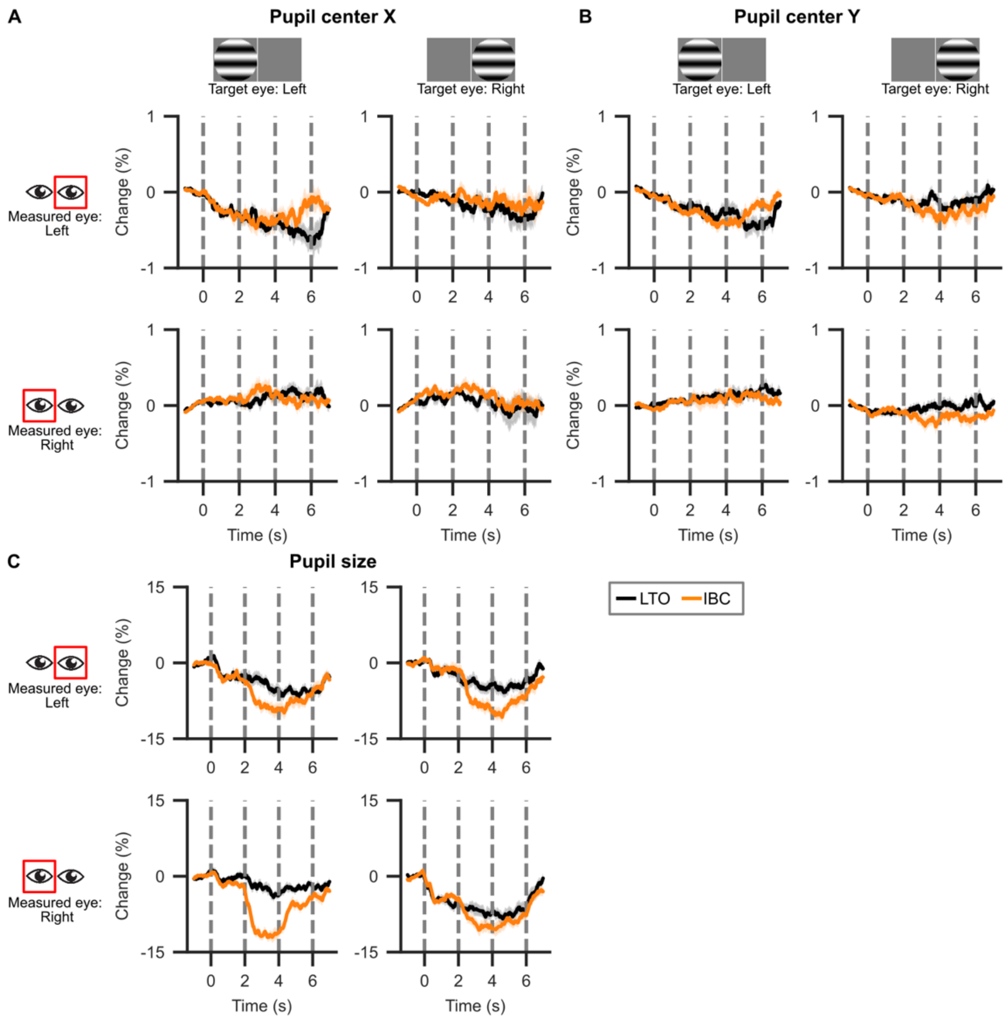


**Supplementary figure 6. Stimulus presentation evokes only minor and symmetric pupil position changes during the awake delayed-onset experiment.**

(A) Change of x-position of pupil center during stimulus presentation compared to x-position during baseline period (1s before stimulus onset). Changes are plotted separately for target displayed on the left or right eye (columns) as well for left and right pupil (rows). Averaged over n = 16 recordings from four mice. Shaded region around lines indicates standard error of the mean. Dashed grey lines indicate stimulus timings (target onset, mask onset, mask offset, target offset). LTO = long target-only, IBC = intermittent binocular conflict.

(B) Like (A) but now for Y-position of pupil center.

(C) Like (A) but now for pupil size.

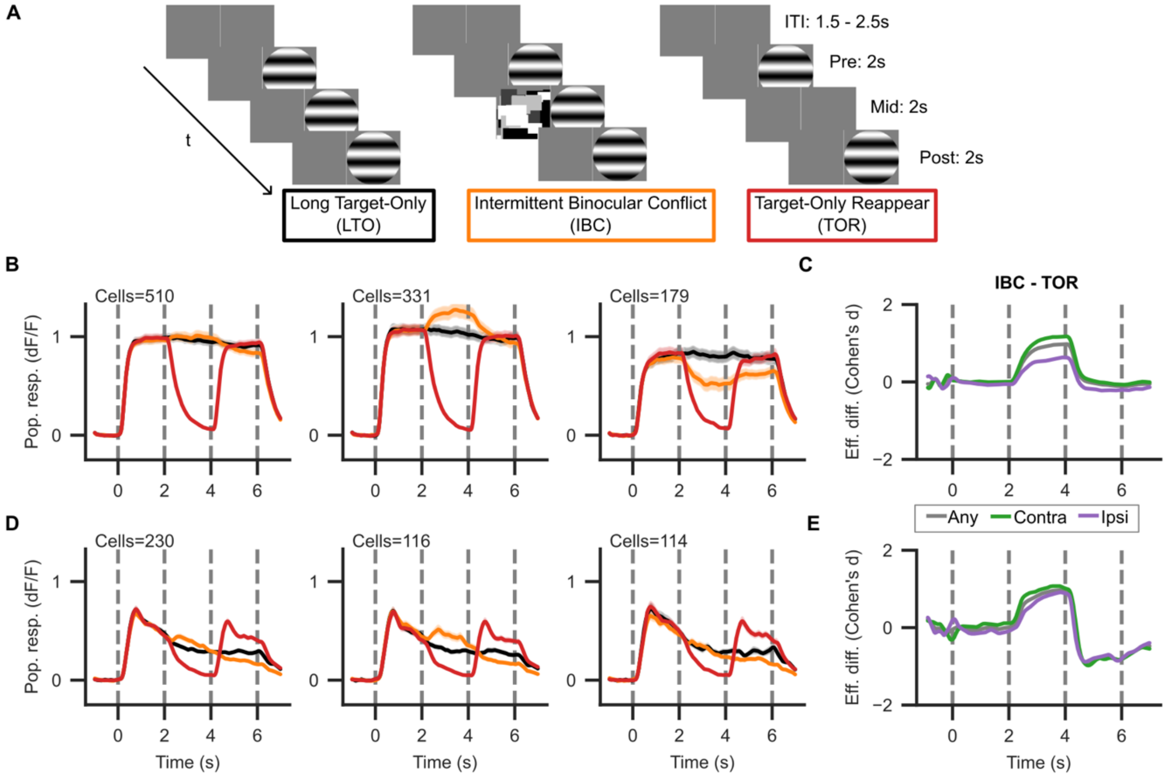


**Supplementary figure 7. Population responses in delayed-onset paradigm with an extra control condition.**

(A) Stimulus paradigm as in Figure 5A but now including an extra control condition: target-only reappear (TOR).

(B) Population responses as in Figure 5D but now including the TOR control condition. The data for LTO and IBC conditions are the same as in Fig.5.

(C) Effective difference (Cohen's d) between IBC and TOR population responses.

(D) Same as B but now under anesthesia.

(E) Same as C but now under anesthesia.

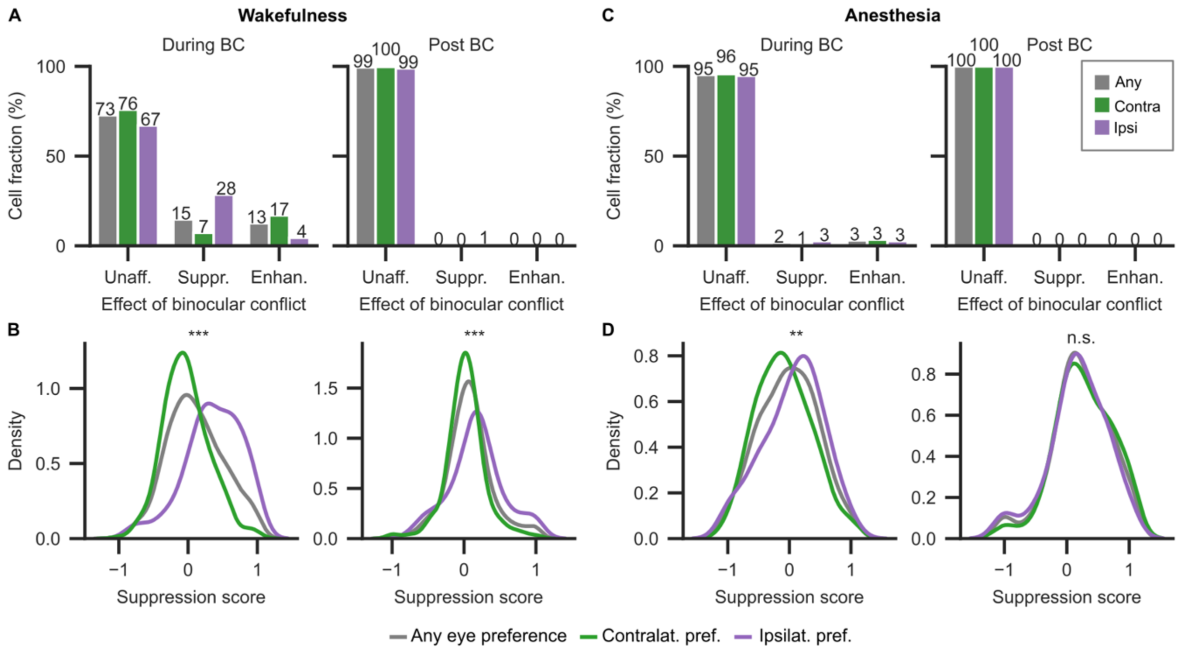


**Supplementary figure 8. Suppression during and after intermittent binocular conflict in the delayed-onset experiment.**

(A) Fractions of significantly modulated cells (n=510; contra-pref: 331, ipsi-pref: 179 cells) during and after intermittent binocular conflict during wakefulness. During binocular conflict: 3 – 4s after target onset, 1-2s after mask onset. After binocular conflict: 5-6s after target onset, 1-2s after mask offset. Significance assessed by two-sided Mann Whitney U test followed by FDR correction. Similar to Figure 3F. Any: all cells, contra: cells whose preferred target was on the contralateral eye, ipsi: cells whose preferred target was on the ipsilateral eye.

(B) Suppression scores of the cells in (A). *** = $p < 0.001$, two-sided Mann Whitney U test between suppression scores of contra- and ipsi-pref cells. Similar to Figure 3E.

(C) Same as A but for cells (n=230; contra-pref: 116, ipsi-pref: 114 cells) recorded during anesthesia.

(D) Same as B but for cells recorded during anesthesia. ** = $p < 0.01$, n.s. = not significant.

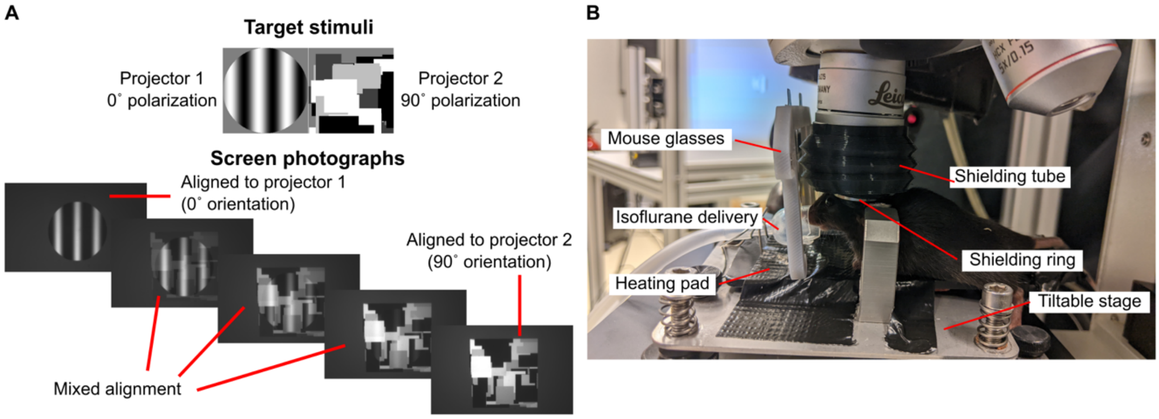


**Supplementary figure 9. Stimulus and recording specifics.**

(A) Demonstration of exclusion through polarization filters. (Top) Schematic representation of binocular stimuli. (Bottom) Photographs through rotating polarization filter to show polarizer alignment.

(B) Photo of anesthetized mouse under microscope with annotated experimental elements.
